# *Oryza sativa* cv. Nipponbare and *Oryza barthii* as Unexpected Tolerance and Susceptibility Sources Against *Schizotetranychus oryzae* (Acari: Tetranychidae) Mite Infestation

**DOI:** 10.3389/fpls.2021.613568

**Published:** 2021-02-10

**Authors:** Giseli Buffon, Édina Aparecida dos Reis Blasi, Thainá Inês Lamb, Janete Mariza Adamski, Joséli Schwambach, Felipe Klein Ricachenevsky, Amanda Bertolazi, Vanildo Silveira, Mara Cristina Barbosa Lopes, Raul Antonio Sperotto

**Affiliations:** ^1^Graduate Program in Biotechnology, University of Taquari Valley-Univates, Lajeado, Brazil; ^2^Biological Sciences and Health Center, University of Taquari Valley-Univates, Lajeado, Brazil; ^3^Graduate Program in Botany, Federal University of Rio Grande do Sul, Porto Alegre, Brazil; ^4^Graduate Program in Biotechnology, University of Caxias do Sul, Caxias do Sul, Brazil; ^5^Graduate Program in Molecular and Cellular Biology, Federal University of Rio Grande do Sul, Porto Alegre, Brazil; ^6^Laboratory of Biotechnology, Bioscience and Biotechnology Center, State University of Northern Rio de Janeiro Darcy Ribeiro, Campos dos Goytacazes, Brazil; ^7^Rice Research Institute (Instituto Rio-Grandense do Arroz), Cachoeirinha, Brazil

**Keywords:** osmotin, proline, protease inhibitors, proteome, resistance, wild species

## Abstract

Cultivated rice (*Oryza sativa* L.) is frequently exposed to multiple stresses, including *Schizotetranychus oryzae* mite infestation. Rice domestication has narrowed the genetic diversity of the species, leading to a wide susceptibility. This work aimed to analyze the response of two African rice species (*Oryza barthii* and *Oryza glaberrima*), weedy rice (*O. sativa* f. *spontanea*), and *O. sativa* cv. Nipponbare to *S. oryzae* infestation. Surprisingly, leaf damage, histochemistry, and chlorophyll concentration/fluorescence indicated that the African species present a higher level of leaf damage, increased accumulation of H_2_O_2_, and lower photosynthetic capacity when compared to *O. sativa* plants under infested conditions. Infestation decreased tiller number, except in Nipponbare, and caused the death of *O. barthii* and *O. glaberrima* plants during the reproductive stage. While infestation did not affect the weight of 1,000 grains in both *O. sativa*, the number of panicles per plant was affected only in *O. sativa* f. *spontanea*, and the percentage of full seeds per panicle and seed length were increased only in Nipponbare. Using proteomic analysis, we identified 195 differentially abundant proteins when comparing susceptible (*O. barthii*) and tolerant (Nipponbare) plants under control and infested conditions. *O. barthii* presents a less abundant antioxidant arsenal and is unable to modulate proteins involved in general metabolism and energy production under infested condition. Nipponbare presents high abundance of detoxification-related proteins, general metabolic processes, and energy production, suggesting that the primary metabolism is maintained more active compared to *O. barthii* under infested condition. Also, under infested conditions, Nipponbare presents higher levels of proline and a greater abundance of defense-related proteins, such as osmotin, ricin B-like lectin, and protease inhibitors (PIs). These differentially abundant proteins can be used as biotechnological tools in breeding programs aiming at increased tolerance to mite infestation.

## Introduction

Rice (*Oryza sativa* L.) is extremely important for human nutrition, representing around 20% of the daily intake calories in the world. It is considered a staple food for over half of the world’s population (Muthayya et al., 2014). To meet this nutritional demand, natural resources must be used efficiently, and crop production needs to be increased (Godfray et al., 2010). However, the search for new agricultural frontiers and the availability of natural resources is limited, exposing rice cultivation to several disturbances caused by biotic and abiotic stresses, which negatively impact grain yield (Palmgren et al., 2015). Among these important stresses that influence plant yield, the presence of arthropods (Oerke, 2006) impacts about 18–26% of annual crop production worldwide, causing losses of over US$ 470 billion (Culliney, 2014). Most of these losses (13–16%) occur in the field before harvesting, and losses are most often described in developing countries (Culliney, 2014). One of the arthropods that attack rice crops in Brazil, and has been reported in other South American countries, is *Schizotetranychus oryzae* Rossi de Simons mite species, which can cause more than 60% loss in rice grain yield (Buffon et al., 2018). An interesting strategy for reducing these losses is the search for mite-tolerant cultivars, as these plants tolerate mite infestation and maintain grain yield without the need to apply acaricides (Sperotto et al., 2018a).

Plant defense to herbivory includes both resistance (the ability of plants to escape attacking enemies) and tolerance (the ability of plants to withstand herbivory without any decline in yield) (Stenberg and Muola, 2017). Both defense strategies can be plant focused and can be useful to measure the plant’s ability to defend from herbivores. Several molecular and physiological characteristics can be used to detect resistance/tolerance plant capacity, including expression of defense-related genes, levels of soluble protein/sugar, reactive oxygen species, lipid peroxidation, carotenoids/chlorophyll, photosynthetic efficiency, cell membrane damage, and others (Blasi et al., 2015).

The search for genetic variability in the *Oryza* genus is a shortcut to obtain resistant/tolerant rice genotypes and to develop cultivars that adapt to natural adversities (Menguer et al., 2017; Szareski et al., 2018). Several authors reported wild rice plants resistant/tolerant to biotic and abiotic factors (Brar and Khush, 2003). The use of wild rice species in breeding programs can facilitate adaptation to biotic and abiotic stresses, as well as meeting the demand for food security in the current scenario of rapidly growing world population (Henry, 2014). The *Oryza* genus is composed of 24 species, two cultivated (*O. sativa* and *Oryza glaberrima*) and other 22 wild species. These wild species represent between 15 and 25 million years of evolutionary diversification (Vaughan, 1994; Menguer et al., 2017).

*Oryza barthii* is an annual wild African species that is commonly known as the progenitor of *O. glaberrima*, the rice cultivated species grown in Africa. *O. barthii* is tolerant to several biotic and abiotic stresses (Khush, 1997), being adapted to more adverse ecological conditions, and resistant to multiple environmental restrictions (Sarla and Swamy, 2005). Weedy rice (*O. sativa* f. *spontanea*), which is the result of rice de-domestication process, also shows tolerance to environmental stresses. Therefore, the genetic background, morphology, and growth behavior are similar to cultivated rice (Qiu et al., 2017). However, differences in tolerance to stressful conditions may occur during de-domestication, e.g., weedy rice is tolerant to cold, high salinity and drought, and resistant to blast disease. Due to its greater stress tolerance/resistance, weedy rice has become one of the most feared and harmful weeds in rice producing regions worldwide (Dai et al., 2014). Nonetheless, interesting traits found in weedy rice can be eventually transferred to cultivated genotypes.

Aiming to identify novel resistance/tolerance mechanisms in rice plants exposed to *S. oryzae* mite infestation, we analyzed two African species (wild *O. barthii* and cultivated *O. glaberrima*), weedy rice (*O. sativa* f. *spontanea*) and cv. Nipponbare (*O. sativa* ssp. *japonica*). Although we expected wild rice species to show higher tolerance to mite infestation, we found unexpected results. According to our data, *O. barthii* and *O. glaberrima* are extremely sensitive to mite infestation, while cv. Nipponbare seems to be more tolerant. Therefore, we tried to understand the molecular and physiological mechanisms behind Nipponbare tolerance and African species susceptibility to this mite. Our results may be useful for future breeding programs aiming at resistance/tolerance to *S. oryzae* mite infestation.

## Materials and Methods

### Plant Growth Conditions and Mite Infestation

Seeds of *O. sativa* cv. Nipponbare (hereafter “Nipponbare”) were obtained from the seed bank Rice Genome Resource Center^1^. Seeds of weedy and African rice species are part of the International Rice Research Institute (IRRI) germplasm collection: *O. sativa* f. *spontanea* (IRGC 80590, hereafter “weedy rice”), *O. barthii* (IRGC 86524), and *O. glaberrima* (IRGC 103959). Seeds were surface sterilized and germinated for 4 days in an incubator (28°C) on paper soaked with distilled water. After germination, plantlets were transferred to vermiculite/soil mixture (1:3) for additional 14 days in greenhouse conditions, and then transferred to plastic buckets containing soil and water. Plastic buckets containing rice plants highly infested by *S. oryzae* were kindly provided by Instituto Rio-Grandense do Arroz (IRGA, Cachoeirinha, RS), and were used to infest rice plants in our experiment. Fifty plants (V10-13 stage, according to Counce et al., 2000) of each cultivar/species (five plants per bucket) were infested by proximity with the bucket containing the highly infested plants placed in the center of the other buckets. For greater homogeneity of infestation and contact, buckets of each cultivar/species were rotated at a 90° angle counterclockwise every 2 days. Ten plants of each cultivar were cultivated without infestation (control condition).

The level of damage caused by *S. oryzae* was analyzed from V10-13 stage until the plants reached the final stage of reproductive development (panicle maturity, R9 stage; Counce et al., 2000). Evaluation of damage in the abaxial and adaxial faces of leaves was based on a classification of four infestation levels: Level 1: control condition, without any sign of infestation; Level 2: early infested (EI) leaves, 10–20% of damaged leaf area, average of 168 h of exposure to the mite; Level 3: intermediate infested (II) leaves, 40–50% of damaged leaf area, average of 360 h; and Level 4: late infested (LI) leaves, more than 80% of damaged leaf area, average of 720 h, according to Buffon et al. (2018).

### Plant Height and Tiller Number

Plant height and tiller number were evaluated (*n* = 50) during the vegetative stage (V10-13, before infestation) and during the last reproductive stage (R9, control and infested plants).

### Chlorophyll *a* Fluorescence Transients

The chlorophyll *a* fluorescence transient was measured (*n* = 10) on the third upper leaves of control and infested plants in two different exposure times, 1 week (which correspond to EI) and 4 weeks (which correspond to LI), using a portable fluorometer (OS30p, Optisciences, United Kingdom). Before the measurements, plants were dark adapted for 20 min, and the fluorescence intensity was measured by applying a saturating pulse of 3,000 μmol photons m^–2^ s^–1^. The resulting fluorescence of the chlorophyll *a* was measured from 0 to 1 s. The chlorophyll fluorescence intensity rises from a minimum level (the O level, F_*O*_ = 160 μs) to a maximum level (the P level, F_*P*_ = 300 ms), via two intermediate steps labeled J (F_*J*_ = 2 ms) and I (F_*I*_ = 30 ms) phases (Stirbet and Govindjee, 2011), also known as OJIP curve (Strasser et al., 2000).

### Total Chlorophyll Concentration

Samples (*n* = 3) of leaves from rice plants submitted to control and LI conditions were collected, and total chlorophyll concentration was quantified. In brief, 100 mg of sample was sprayed in liquid nitrogen, homogenized in 1.5 mL of 85% (v/v) acetone, maintained in the dark for 30 min, and centrifuged at 12,000 *g* for 3 min. The supernatant was transferred to a new tube, and the process was repeated until complete discoloration of the plant tissue. Chlorophyll *a* and *b* were quantified by measuring absorbance at 663 and 645 nm, and the concentrations were calculated according to Ross (1974).

### *In situ* Histochemical Localization of H_2_O_2_

*In situ* accumulation of H_2_O_2_ in control and LI leaves was detected by histochemical staining with DAB, according to Shi et al. (2010), with minor modifications. Leaves were excised and immersed in DAB solution (1 mg mL^–1^, pH 3.8) in 10 mM phosphate buffer (pH 7.8) and incubated at room temperature for 8 h in the light until brown spots were visible, which are derived from the reaction of DAB with H_2_O_2_. Leaves were bleached in boiling concentrated ethanol to visualize the brown spots, which were kept in 70% ethanol for taking pictures with a digital camera coupled to a stereomicroscope.

### Seed Analysis

Seeds of Nipponbare and weedy rice were collected in R9 stage, and the following agronomical parameters were evaluated: number of panicles per plant, number of seeds (empty + full) per panicle, percentage of full seeds per panicle, grain length, and weight of 1,000 full grains. Yield reduction caused by *S. oryzae* infestation in Nipponbare was calculated using the following equation, for each condition (control and infested): number of seeds (empty + full) per plant × percentage of full seeds × weight of one seed = seed weight per plant. Seeds from African rice species were not analyzed as these plants did not reach the reproductive stage.

### Proline Quantification

Control and LI leaves of Nipponbare and weedy rice were collected, and proline accumulation was quantified (*n* = 3) according to Shukla et al. (2012). In brief, 250 mg of sample was sprayed in liquid nitrogen, homogenized in 2 mL of sulfosalicylic acid (3%), and centrifuged at 10,000 *g* for 30 min. Then, 1 mL of the supernatant was transferred to a new tube, and 1 mL of glacial acetic acid and 1 mL of ninhydrin (1.25 g of ninhydrin + 30 mL of glacial acetic acid + 20 mL of 6 M phosphoric acid) were added. The homogenized mixture was boiled in a water bath at 100°C for 30 min. Subsequently, the reaction was cooled on ice and 2 mL of toluene was added, mixing in the vortex for 30 s. The upper phase containing proline was measured on the spectrophotometer at 520 nm. The proline level (mmol g^–1^ fresh weight) was quantified using L-proline as standard. The standard curve was prepared from a stock solution of proline (5 mM) in sulfosalicylic acid for a concentration range between 10 and 320 mmol mL^–1^. The free proline concentration of each sample was calculated from the linear regression equation obtained from the standard curve. The procedure for constructing the standard curve was the same as for free proline determination of the samples.

### Plant Protein Extraction and Quantification

Three biological samples (250 mg of fresh weight) of control and EI leaves from *O. barthii* (mite-sensitive) and Nipponbare (mite-tolerant), each containing three leaves from three different plants, were subjected to protein extraction using Plant Total Protein Extraction Kit (Sigma–Aldrich). The protein concentration was measured using 2-D Quant Kit (GE Healthcare, Piscataway, NJ, United States).

### Protein Digestion

For protein digestion, three biological replicates of 100 μg of proteins from *O. barthii* and Nipponbare leaves were used. Before the trypsin digestion step, protein samples were precipitated using the methanol/chloroform methodology to remove any detergent from samples (Nanjo et al., 2012), followed by resuspension in urea 7 M and thiourea 2 M buffer in 50 mM ammonium bicarbonate. Right after, protein digestion was performed using the filter-aided sample preparation (FASP) methodology (Wiśniewski et al., 2009), with modifications described by Burrieza et al. (2019). In brief, after checking the integrity of the Microcon-30 kDa (Merck Millipore, Germany) filter units, protein aliquots were added to the filter units, washed with 200 μL of 50 mM ammonium bicarbonate (solution A), and centrifuged at 10,000 *g* for 15 min at 25°C (unless otherwise stated, all centrifugation steps were performed under this condition). This step was repeated once for complete removal of urea before reduction of proteins. Next, 100 μL of 50 mM DTT (GE Healthcare), freshly made in solution A, was added, gently vortexed, and incubated for 20 min at 60°C (1 min agitation and 4 min resting, at 650 r/min). Then, 200 μL of 8 M urea in 50 mM ammonium bicarbonate (solution B) was added, and centrifuged for 15 min. For protein alkylation, 100 μL of 50 mM iodoacetamide (GE Healthcare), freshly prepared in solution B, was added, gently vortexed, and incubated for 20 min at 25°C in the dark (1 min agitation and 19 min resting, at 650 r/min). Next, 200 μL of solution B was added and centrifuged for 15 min. This step was repeated once. Then, 200 μL of solution A was added and centrifuged for 15 min. This step was repeated twice. Approximately 50 μL of the sample should remain in the last washing. For protein digestion, 25 μL of 0.2% (v/v) RapiGest (Waters, Milford, CT, United States) and 25 μL of trypsin solution (1:100 enzyme:protein, V5111, Promega, Madison, WI, United States) were added, gently vortexed, and incubated for 18 h at 37°C (1 min agitation and 4 min resting, at 650 r/min). For peptide elution, the filter units were transferred to new microtubes and centrifuged for 10 min. Then, 50 μL of solution A was added and centrifuged for 15 min. This step was repeated once. For RapiGest precipitation and trypsin inhibition, 5 μL of 15% trifluoroacetic acid (Sigma–Aldrich) was added, gently vortexed, and incubated for 30 min at 37°C. Then, samples were centrifuged for 15 min, and the supernatants collected and vacuum dried. Peptides were resuspended in 100 μL solution of 95% 50 mM ammonium bicarbonate, 5% acetonitrile, and 0.1% formic acid. The resulting peptides were quantified by the A205 nm protein and peptide methodology, using a NanoDrop 2000c spectrophotometer (Thermo Fisher Scientific).

### Mass Spectrometry Analysis

A nanoAcquity UPLC connected to a Synapt G2-Si HDMS mass spectrometer (Waters, Manchester, United Kingdom) was used for ESI-LC-MS/MS analysis. Runs consisted of injecting 1 μg of digested peptides from each biological replicate. During separation, samples were loaded onto the nanoAcquity UPLC 5 μm C18 trap column (180 μm × 20 mm) at 5 μL/min during 3 min and then onto the nanoAcquity HSS T3 1.8 μm analytical reversed phase column (75 μm × 150 mm) at 400 nL/min, with a column temperature of 45°C. For peptide elution, a binary gradient was used, with mobile phase A consisting of water (Tedia, Fairfield, OH, United States) and 0.1% formic acid (Sigma–Aldrich), and mobile phase B consisting of acetonitrile (Sigma–Aldrich) and 0.1% formic acid. Gradient elution started at 7% B, then ramped from 7% B to 40% B up to 91.12 min, and from 40% B to 99.9% B until 92.72 min, being maintained at 99.9% until 106.00 min, then decreasing to 7% B until 106.1 min and kept 7% B until the end of the experiment at 120.00 min. Mass spectrometry was performed in positive and resolution mode (V mode), 35,000 FWHM, with ion mobility, and in data-independent acquisition (DIA) mode; ion mobility separation (HDMS^*E*^) using IMS wave velocity of 600 m/s, and helium and IMS gas flow of 180 and 90 mL/min, respectively; the transfer collision energy ramped from 19 V to 55 V in high-energy mode; cone and capillary voltages of 30 and 2750 V, respectively; and a source temperature of 70°C. In TOF parameters, the scan time was set to 0.5 s in continuum mode with a mass range of 50–2,000 Da. The human [Glu1]-fibrinopeptide B (Sigma–Aldrich) at 100 fmol/μL was used as an external calibrant and lock mass acquisition was performed every 30 s. Mass spectra acquisition was performed by MassLynx v4.0 software.

### Proteomics Data Analysis

Spectral processing and database searching conditions were performed by ProteinLynx global server (PLGS; version 3.0.2) (Waters) and ISOQuant workflow software (Distler et al., 2014). The analysis used the following parameters: Apex3D of 150 counts for low energy threshold, 50 counts for elevated energy threshold, and 750 counts for intensity threshold; one missed cleavage, minimum fragment ion per peptide equal to three, minimum fragment ion per protein equal to seven, minimum peptide per protein equal to two, fixed modifications of carbamidomethyl (C), and variable modifications of oxidation (M) and phosphoryl (STY). The false discovery rate (FDR) for peptide and protein was set to a maximum of 1%, with a minimum peptide length of six amino acids. The analysis used the *O. sativa* ssp. *japonica* reference proteome from Uniprot^2^ (48,903 protein sequences downloaded on 18 August 2018).

Comparative label-free quantification analysis was performed using ISOQuant software (Distler et al., 2014). Briefly, the analysis included retention time alignment, exact mass retention time (EMRT) and IMS clustering, as well as data normalization and protein homology filtering. ISOQuant annotates the resulting feature clusters by evaluating consensus peptide identifications and identification probabilities. Protein identification parameters in ISOQuant were set to FDR 1%, peptide score greater than six, minimum peptide length of six amino acids, and at least two peptides per protein. Label-free quantification was estimated using the TOP3 quantification approach (Silva et al., 2006), followed by the multidimensional normalization process implemented within ISOQuant (Distler et al., 2014). After data processing and to ensure the quality of results, only proteins present or absent (for unique proteins) in three out of three runs were accepted and submitted to differential abundance analysis. Data were analyzed using Student’s *t*-test (two-tailed). Proteins with *P* < 0.05 were considered more abundant if the Log_2_ of fold change (FC) was greater than 0.5 and less abundant if the Log_2_ of FC was less than −0.5. All proteomics data were deposited to the ProteomeXchange Consortium (Deutsch et al., 2017) via the PRIDE repository^3^ (Perez-Riverol et al., 2019) and can be accessed using the code PXD020940.

### Statistical Analysis

Except for proteomics, data from other experiments were analyzed using the Student’s *t*-test (*P* ≤ 0.05 and 0.01) or one-way ANOVA followed by Tukey test (*P* ≤ 0.05), using SPSS Base 23.0 for Windows (SPSS Inc., United States).

## Results

### Different Responses of Rice Cultivars/Species to *S. oryzae* Infestation

**FIGURE 1:**
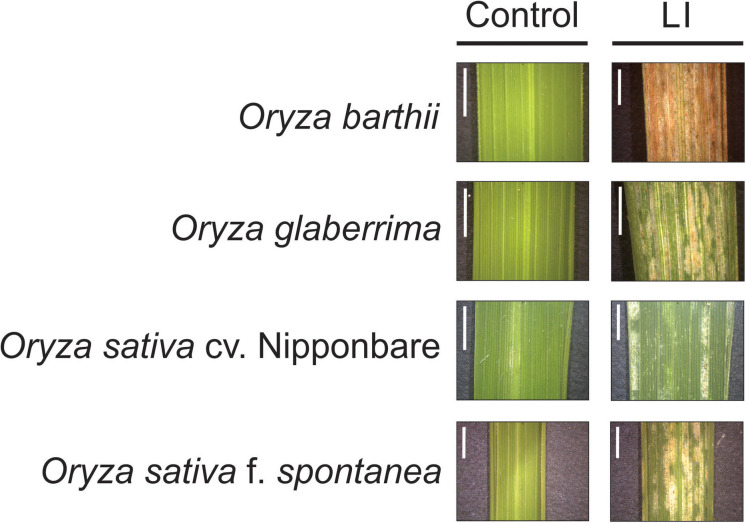
Visual characteristics of leaves from control and late infested (LI) plants of *Oryza barthii*, *Oryza glaberrima*, *Oryza sativa* cv. Nipponbare, and weedy rice (*Oryza sativa* f. *spontanea*). Bars indicate 0.5 cm.

**FIGURE 2:**
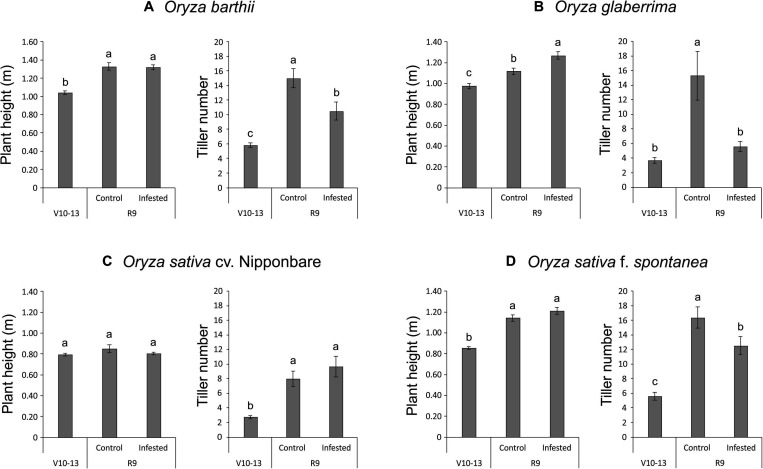
Plant height (m) and tiller number of *Oryza barthii* **(A)**, *Oryza glaberrima* **(B)**, *Oryza sativa* cv. Nipponbare **(C)**, and weedy rice (*Oryza sativa* f. *spontanea*) **(D)** at the vegetative stage (no infestation, V10-13) and full maturity stage (control or infested conditions, R9). Represented values are the averages of 50 samples ± SE. Different letters indicate that the means are different by the Tukey HSD test (*P* ≤ 0.05).

Unexpectedly, we verified that African rice species (*O. barthii* and *O. glaberrima*) and weedy rice (*O. sativa* f. *spontanea*) presented high susceptibility to *S. oryzae* infestation, while *O. sativa* cv. Nipponbare showed low levels of leaf damage (Figure 1). After 4 weeks of infestation, leaves of *O. barthii* were dry and the leaf area was totally injured. The species *O. glaberrima* presented infestation level 4 (above 80% of the damaged leaf area), while Nipponbare presented level 2 (10–20% of damaged leaf area), and weedy rice revealed level 3 (40–50% of the damaged leaf area), as shown in Figure 1. Both African species died before reaching the reproductive stage, while both *O. sativa* were able to complete their life cycle and set seeds. Although the plant height was not negatively affected by mite infestation in any of the tested cultivars/species, tiller number decreased under infested conditions in all plants, except in Nipponbare (Figure 2).

**FIGURE 3:**
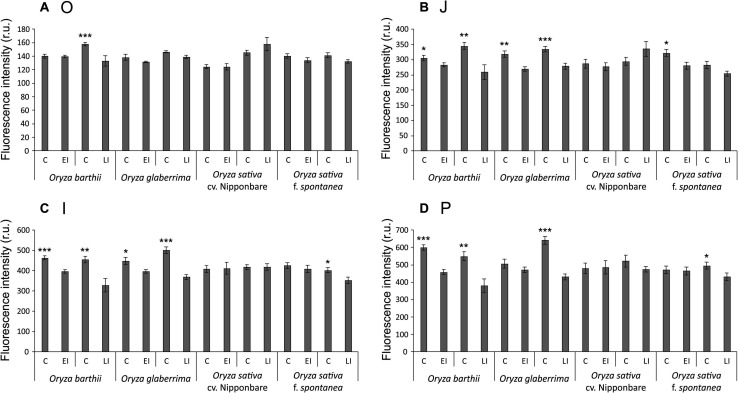
OJIP-test parameters calculated from the chlorophyll *a* fluorescence transient in control (C), early infested (EI), and late infested (LI) leaves of *Oryza barthii*, *Oryza glaberrima*, *Oryza sativa* cv. Nipponbare, and weedy rice (*Oryza sativa* f. *spontanea*). **(A)** O; **(B)** J; **(C)** I; **(D)** P. Represented values are the averages of 10 samples ± SE. Mean values (from each species/cultivar and each exposure time: C × EI; C × LI) with one, two, or three asterisks are significantly different as determined by a Student’s *t*-test (*P* ≤ 0.05, 0.01, and 0.001, respectively). r.u., Raman units.

**FIGURE 4:**
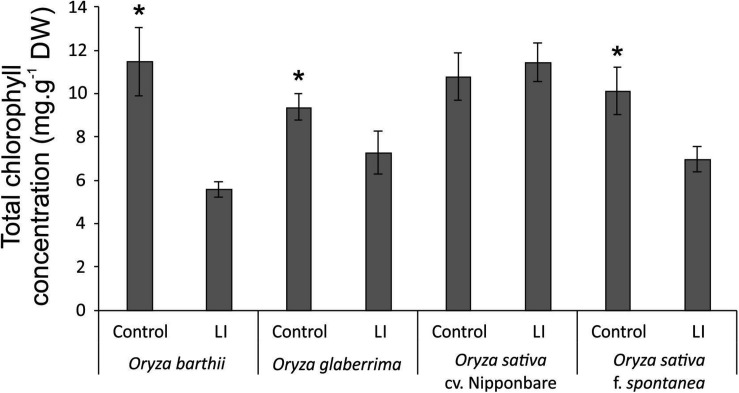
Total chlorophyll concentration in control and late infested (LI) leaves of *Oryza barthii*, *Oryza glaberrima*, *Oryza sativa* cv. Nipponbare, and weedy rice (*Oryza sativa* f. *spontanea*) plants. Represented values are the averages of three samples ± SE. Mean values (from each species/cultivar: C × LI) with one asterisk are significantly different as determined by a Student’s *t*-test (*p*-value ≤ 0.05). DW, dry weight.

**FIGURE 5:**
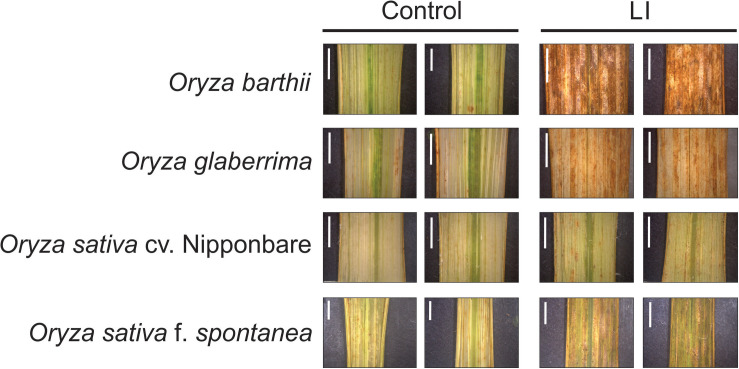
Histochemical staining assay of H_2_O_2_ by diaminobenzidine (DAB) in control and late infested (LI) leaves of *Oryza barthii*, *Oryza glaberrima*, *Oryza sativa* cv. Nipponbare, and weedy rice (*Oryza sativa* f. *spontanea*). The positive staining (detected in higher levels on infested leaves) in the photomicrographs shows as bright images (brown-color). Bars indicate 0.5 cm.

Chlorophyll *a* fluorescence analysis showed that African rice species (*O. barthii* and *O. glaberrima*) and weedy rice presented a decrease in at least one of the OJIP curve-times. *O. barthii* seems to be the most affected species, with a decrease in all curve-times after four weeks of infestation (LI leaves), while *O. glaberrima* presented a decrease in J-I-P stages, and weedy rice in I-P stages. Nipponbare was the only one with no decrease during the entire OJIP curve under infested condition (Figure 3), suggesting that mite infestation triggers lower damage in Nipponbare photosynthetic apparatus than in the other tested cultivars/species. These results agree with total chlorophyll concentration analysis, where Nipponbare was the only tested cultivars/species with no decrease under infested conditions, while *O. barthii* presented the highest decrease comparing infested and control conditions (Figure 4). Also, histochemical staining assay using DAB indicated that Nipponbare leaves accumulate lower levels of H_2_O_2_ than the other tested cultivars/species, especially when compared to *O. barthii* (Figure 5). Therefore, *S. oryzae* infestation differentially affects the generation of oxidative stress in LI leaves of the tested plants. Altogether, we suggest that *O. barthii* is extremely susceptible to *S. oryzae* infestation, while Nipponbare can be considered tolerant.

**FIGURE 6:**
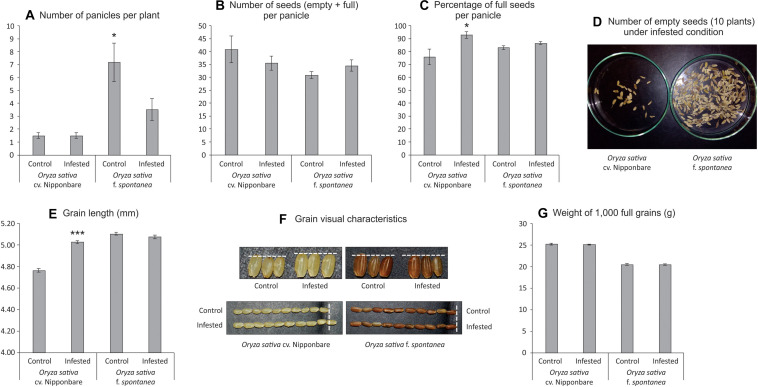
Seeds analysis of *Oryza sativa* cv. Nipponbare and weedy rice (*Oryza sativa* f. *spontanea*). **(A)** Number of panicles per plant. **(B)** Number of seeds (empty + full) per panicle. **(C)** Percentage of full seeds per panicle. **(D)** Number of empty seeds (10 plants) under infested condition. **(E)** Grain length (mm). **(F)** Grain visual characteristics. **(G)** Weight of 1,000 full grains. Represented values are the averages of 50 samples ± SE. Mean values (from each cultivar: Control × Infested) with one or three asterisks are significantly different as determined by a Student’s *t*-test (*P* ≤ 0.05 and 0.001).

**FIGURE 7:**
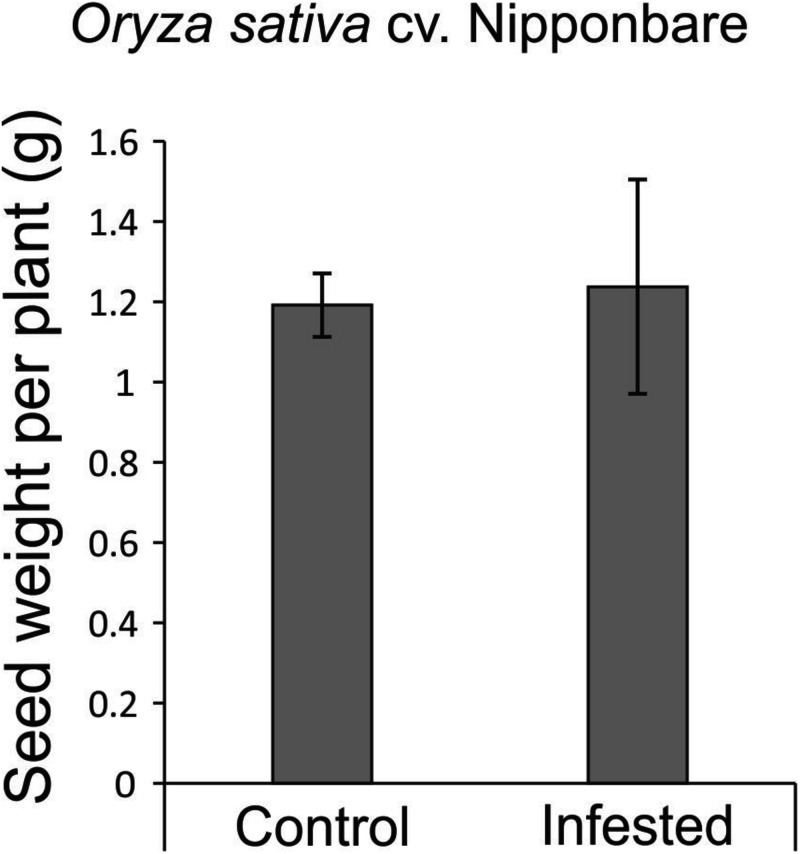
Seed weight per plant (estimative of yield) of *Oryza sativa* cv. Nipponbare under control and infested conditions. Represented values are the averages of 10 samples ± SE.

As African rice species were not able to reach the reproductive stage, seeds from Nipponbare and weedy rice were evaluated to verify whether *S. oryzae* infestation could impact seed production. Weedy rice presents higher number of panicles per plant than Nipponbare under both control and infested conditions (Figure 6A), even though the number of panicles per plant was affected by *S. oryzae* infestation only in weedy rice (Figure 6A). In Nipponbare, none of the analyzed parameters (number of panicles per plant— Figure 6A, number of seeds per panicle—Figure 6B, percentage of full seeds per panicle—Figure 6C, grain length—Figures 6E,F, and weight of 1,000 full grains—Figure 6G) was negatively affected by mite infestation. As seen in Figure 6C, 93% of the seeds produced by Nipponbare plants under infested condition were full, while in the same condition, weedy rice produced 87% of full seeds. Such difference, together with the higher number of panicles per plant in weedy rice than Nipponbare under infested condition (Figure 6A), probably explains the high number of empty seeds in weedy rice plants infested by *S. oryzae* (Figure 6D). Surprisingly, two parameters (percentage of full seeds per panicle—Figure 6C and grain length—Figures 6E,F) presented higher values in Nipponbare plants under infested than control condition, resulting in the maintenance of seed weight per plant (an estimate of yield) under infested condition (Figure 7). Therefore, Nipponbare reveals physiological characteristics of tolerance, since even during infestation, plants can maintain development, photosynthetic/antioxidant activities, and grain yield.

### Overview of Proteomic Analysis

**FIGURE 8:**
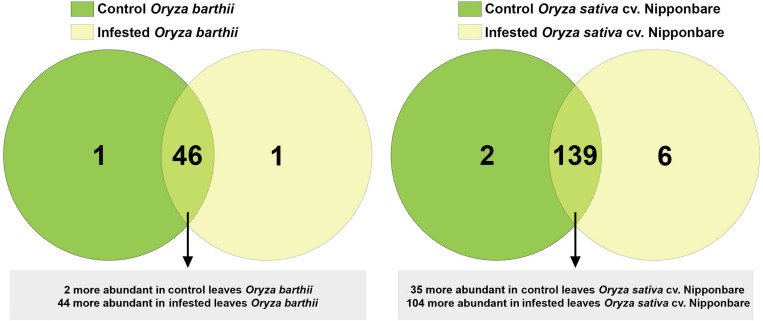
Venn diagram showing the overlap of rice proteins identified in control and early infested (EI) leaves of mite susceptible *Oryza barthii* and mite-tolerant *Oryza sativa* cv. Nipponbare. Dark green circles: control leaves; yellow circles: EI leaves. Light green means overlap.

A total of 1,041 proteins were identified comparing control and infested conditions in *O. barthii* and Nipponbare, with 195 (18.7%) proteins unique to or differentially abundant between treatments. As seen in Figure 8, comparing control and infested leaves of *O. barthii*, we detected 48 differentially abundant proteins, being two more abundant (and one unique) in control condition, and 44 more abundant (and one unique) in infested condition. We identified 147 differentially abundant proteins in control and infested conditions of Nipponbare, being 35 more abundant (and two unique) in control condition, and 104 more abundant (and six unique) in infested condition. Due to the different genetic background between *O. barthii* and *O. sativa*, we prefer not to compare the same conditions between species, in order to avoid the emergence of species-specific sequences that are not mite related.

The corresponding sequence of each identified protein was compared to NCBI BLASTp to identify specific domains, molecular functions, and protein annotations. Afterward, proteins were arbitrarily categorized in functional categories, according to the available literature and their putative molecular function. The lists of all unique or differentially abundant proteins identified in this work are presented in Supplementary Tables 1, 2. In order to facilitate the overall understanding of the differential protein abundance in each functional category presented in Supplementary Tables 1, 2, we summarize these data in Table 1. We detected four different patterns, based on the number of differentially abundant proteins: (1) most of the functional categories (antioxidant system, carbohydrate metabolism/energy production, general metabolic processes, hormone-related, lipid metabolism, protein modification/degradation, stress response, and others) were more represented in infested conditions, regardless the analyzed species; (2) four categories were differentially represented only in one species (amino acid metabolism in *O. barthii* under infested condition; cytoskeleton and transport in Nipponbare under control condition; and protease inhibitor (PI) also in Nipponbare, but under infested condition); (3) photosynthesis (over represented in *O. barthii* under infested condition, and in Nipponbare under control condition); and (4) translation, with no difference between control and infested conditions, regardless of the analyzed species.

**TABLE 1:**
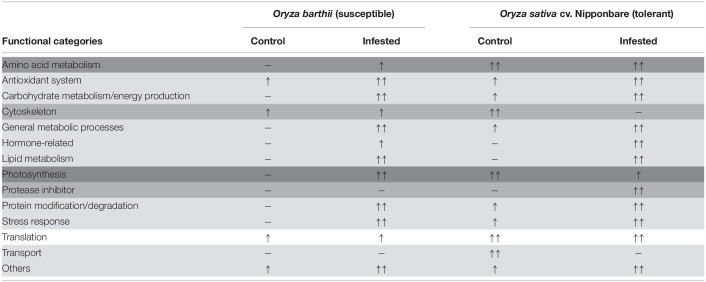
Schematic representation of the functional categories differently represented in leaves of mite susceptible *Oryza barthii* and mite-tolerant *Oryza sativa* cv. Nipponbare plants under control and infested conditions.

*Different shades of gray color represent the different patterns of response. Number of arrows (one or two) relates to the representation level, which is based on the number of differentially abundant sequences. (−): functional category not detected as differentially represented.*

**FIGURE 9:**
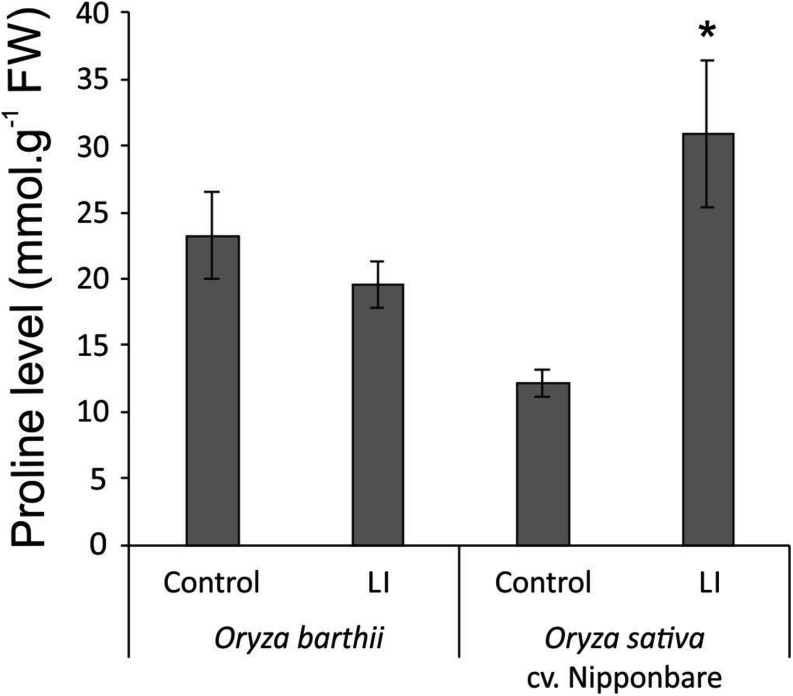
Proline levels in control and late infested (LI) leaves of mite susceptible *Oryza barthii* and mite-tolerant *Oryza sativa* cv. Nipponbare. Represented values are the averages of three samples ± SE. Mean values with one asterisk are significantly different as determined by a Student’s *t*-test (*P* ≤ 0.05). FW, fresh weight.

Considering the tolerant Nipponbare, *S. oryzae* infestation seems to be less damaging and to generate a more complex defense response. It is interesting to highlight the higher diversity and expression level of antioxidant proteins in Nipponbare than *O. barthii* under infested conditions, which agrees with the lower oxidative stress seen in Nipponbare leaves (Figure 5), and also the higher diversity of proteins involved in general metabolic processes and carbohydrate metabolism/energy production, suggesting that Nipponbare can maintain the basal/primary metabolism more active than *O. barthii* under infested condition. This finding also agrees with our observation that photosynthesis is maintained in Nipponbare, whereas it is drastically affected in *O. barthii*. At the same time, Nipponbare seems more able to fight *S. oryzae* infestation than *O. barthii*, presenting higher diversity and/or representation level of PIs and proteins involved in stress response. As seen in Figure 9, we also detected enhanced proline levels only in Nipponbare leaves under infested condition, suggesting an active defense mechanism. Several proteins belonging to these functional categories are discussed in more detail below.

## Discussion

Our first screening of *Oryza* cultivars/species responses to *S. oryzae* infestation showed that none presents a classical resistance response involving antibiosis and antixenosis (Stenberg and Muola, 2017), since mite population increased over time for all cultivars/species, even though with different kinetics (Figure 1). Physiological analysis and agronomical parameters showed that *O. glaberrima* and especially *O. barthii* are extremely susceptible to *S. oryzae* infestation (being unable to reach the reproductive stage), while weedy rice and especially Nipponbare are less affected by mite infestation (Figures 1–7). Recently, we detected an *indica* rice cultivar able to maintain seed yield under *S. oryzae* infestation condition, which we characterized as mite tolerant (Buffon et al., 2018). Even though wild plant species have been widely recognized as valuable source of resistance genes for developing herbivore-resistant cultivars, Veasey et al. (2008) and Chandrasena et al. (2016) found no signs of mite resistance when different wild rice species were, respectively, tested against the infestation of *S. oryzae* and *Steneotarsonemus spinki*. These data differ from previously described ones, where *O. barthii* and *O. glaberrima* are recognized as resistant to diseases and pests (Khush, 1997; Linares, 2002). Still, our report is one of the first to test mite tolerance in wild and cultivated rice species.

Grain size is an important component trait of grain yield, which is frequently threatened by stress conditions (Lv et al., 2019; Dong et al., 2020). Interestingly, in our analysis, Nipponbare plants under infested condition presented higher seed length when compared to control condition (Figures 6E,F). Several studies have identified key grain size regulator genes, involved in different mechanisms (reviewed by Li et al., 2018). Even though none of the 43 genes cited by Li et al. (2018) as involved in rice grain size control code proteins differentially abundant in our proteomic analysis, it would be interesting to go deeper in such question, since we found similar results in a previous study with another mite-tolerant *indica* rice cultivar (IRGA 423—Buffon et al., 2018). Recent studies have identified molecular links between regulation of grain size and abiotic stress tolerance (Chen et al., 2016, 2019; Lv et al., 2019; Dong et al., 2020). However, as far as we know, any link between grain size regulation and biotic stress tolerance is unknown.

Recently, Sperotto et al. (2018b) hypothesized that mite-sensitivity presented by wild rice species could be explained, at least partially, by a presumable high GA:JA ratio in these plants, since most of the wild rice species are tall plants and probably present high GA levels. The roles of GA-JA in growth-defense conflicts during herbivory are not yet fully characterized. However, it is already known that plants need to prioritize GA- or JA-induced responses (Yang et al., 2019). Even though proteins related to GA biosynthesis have not been detected as differentially abundant in *O. barthii* or Nipponbare under infested conditions (Supplementary Tables 1, 2), the height of these plants at full maturity stage is quite different (55% higher in *O. barthii*—Figure 2). It is important to highlight that *O. barthii* and Nipponbare plant heights are not affected by *S. oryzae* infestation (Figure 2), and this could explain the lack of differentially abundant proteins involved in GA biosynthesis when we compare control and infested conditions. Sperotto et al. (2018b) suggest short *Oryza* species (*O. minuta*, *O. meyeriana*, *O. neocaledonica*, and *O. schlechteri*) as primary sources of herbivory tolerance, including mites. According to these authors, plants with low GA level/sensitivity would probably amplify JA responses and drive plant resources toward defense instead of growth. Based on our data, the GA-based mechanism is not involved in the different tolerance level found in the tested species, although JA might play a role.

One of the most abundant proteins found in Nipponbare under infested condition is lipoxygenase (log2 FC 4.7), which is well known as a precursor of plant defense mechanisms. Once the herbivore feeds on plant tissues, polyunsaturated fatty acids are released from cell membranes, and accumulate at the wound site (Roach et al., 2015). Also, polyunsaturated fatty acids act as a substrate to lipoxygenase, leading to the production of several defense-related compounds, including different structures of JAs such as methyl jasmonate (MeJA) (Mochizuki et al., 2016; Yang et al., 2019). We detected a key enzyme related to JA biosynthesis, allene oxide cyclase (AOC), which converts allene oxide to 12-oxophytodienoic acid (Nguyen et al., 2020), more abundant in both species under infested conditions. However, the log2 FC is higher in Nipponbare than *O. barthii* (Supplementary Tables 1, 2). Therefore, it is possible that Nipponbare presents a more active JA biosynthesis/signaling pathway, which can probably contribute to mite tolerance. Interestingly, MYB and WRKY transcription factors, which are involved in the activation of defense responses in plants, including JA signaling (Wang et al., 2014; Zhou and Memelink, 2016), were not detected as differentially abundant in our work, raising the possibility of new signaling/defense pathways in *O. barthii* and Nipponbare infested by *S. oryzae*.

The functional category most affected by *S. oryzae* infestation was antioxidant system, with 10 proteins more abundant in the mite susceptible *O. barthii* (Supplementary Table 1), and 22 in the mite-tolerant Nipponbare (Supplementary Table 2) under infested condition. This indicates that both species try to defend and maintain its redox homeostasis, since it is governed by the presence of large antioxidant pools that absorb and buffer reductants and oxidants (Foyer and Noctor, 2005). Considering the leaf damage (Figure 1), H_2_O_2_ accumulation (Figure 5), and the high diversity of differentially abundant proteins (Supplementary Tables 1, 2), such strategy seems to be more efficient in the mite-tolerant Nipponbare than in *O. barthii*. Six proteins identified as more abundant under infested condition only in Nipponbare are ferredoxin, ferredoxin-NADP^+^ reductase, thioredoxin H-type, peroxiredoxin, glutathione reductase, and superoxide dismutase (Supplementary Table 2). The stromal ferredoxin–thioredoxin system, a regulatory mechanism linking light to the activity of associated enzymes, is the best-characterized redox signal transduction system in plants, functioning in the regulation of photosynthetic carbon metabolism (Foyer and Noctor, 2005; Schürmann and Buchanan, 2008). Ferredoxin-NADP^+^ reductase catalyzes the reduction of NADP^+^ to NADPH, using the electrons provided by reduced ferredoxin (Cassan et al., 2005). Both NADPH and reduced ferredoxin are required for reductive assimilation and light/dark activation/deactivation of enzymes. Ferredoxin-NADP^+^ reductase is therefore a hub, connecting photosynthetic electron transport to chloroplast redox metabolism (Kozuleva et al., 2016). Even though the precise mechanism remains unknown, a clear correlation between ferredoxin-NADP^+^ reductase content and tolerance to oxidative stress is well established (Giró et al., 2006; Kozuleva et al., 2016).

Thioredoxins H-type are involved in the cellular protection against oxidative stress (Gelhaye et al., 2004), and function as electron donors to several antioxidant enzymes, such as peroxiredoxin and glutathione reductase (Rouhier et al., 2001). Plants over-expressing thioredoxin H-type genes show oxidative stress tolerance and lower levels of H_2_O_2_ accumulation (Sun et al., 2010; Zhang et al., 2011), similar to our findings with the mite-tolerant Nipponbare (Figure 5). Peroxiredoxins are abundant low-efficiency peroxidases that act as antioxidant and are involved in modulating redox-dependent signaling cascades (Dietz, 2003). Also, after being reduced by thioredoxin, peroxiredoxins reductively convert H_2_O_2_ to H_2_O at the chloroplast (König et al., 2002), acting together with other peroxidase enzymes that use ascorbate as the electron donor. Ascorbate can be directly regenerated using photosynthetic electrons via reduced ferredoxin or using reduced glutathione (GSH). In the chloroplast, the oxidized glutathione (GSSG) is reduced by the glutathione reductase enzyme using NADPH (Kozuleva et al., 2016). It is important to highlight that very efficient scavenging mechanisms exist in chloroplasts to prevent oxidative damage, including the rapid conversion of superoxide radical (O_2_^•–^, one of the dominant species produced during light excitation) to H_2_O_2_ by superoxide dismutase enzymes (Mubarakshina and Ivanov, 2010). This is the most likely explanation for the lack of differences in O_2_^•–^ accumulation in the leaves of plants subjected to control and infested conditions (data not shown). Altogether, these data show that the mite-tolerant Nipponbare under infested condition can maintain the correct balance between rapid removal of damaging oxidative species and appropriate concentrations necessary to initiate signaling cascades.

We verified that all tested cultivars/species revealed a decrease in chlorophyll fluorescence parameters and total chlorophyll concentration, except the mite-tolerant Nipponbare (Figures 3, 4). Increased photosynthesis is an important mechanism behind tolerance to herbivory (Thomson et al., 2003), and parameters of chlorophyll fluorescence (excess energy absorbed by chlorophyll that is reemitted as light) have been used to obtain quantitative information on the photosynthetic performance of plants (Maxwell and Johnson, 2000), since chlorophyll fluorescence curves allow to evaluate the physiological condition of photosystem II (PSII) components and photosynthetic electron transport chain (Kalaji et al., 2016), where ferredoxin protein acts. Recently, Madriaza et al. (2019) indicated that chlorophyll fluorescence is an accurate predictor of tolerance to herbivore damage, and claimed that this rapid methodology could be used to estimate plant tolerance to herbivory in natural populations.

Even though plant height was not affected by *S. oryzae* infestation in *O. barthii* and Nipponbare, the mite susceptible *O. barthii* presented a decrease in tiller number under infested condition, while Nipponbare maintained the same level found in control condition (Figure 2), resulting in no yield losses (Figure 7). Reduction in tillering (and consequently in grain weight) was already seen in rice plants infested by the rice water weevil, *Lissorhoptrus oryzophilus* Kuschel (Zou et al., 2004), and also in IR22 (a rice variety susceptible to the brown planthopper, *Nilaparvata lugens*) (Horgan et al., 2018). It is important to highlight that planthopper damage to IR22 in field cages was severe, and plant death occurred in most of IR22 plants, similar to our findings with *O. barthii* and *O. glaberrima* under infested conditions. Both tillering and seed filling processes are expensive for the crop energy balance. As seen in Supplementary Tables 1, 2, we verified a larger diversity of proteins involved in general metabolic processes and carbohydrate metabolism/energy production under infested condition in tolerant Nipponbare than in susceptible *O. barthii*, suggesting that general metabolism and energy production in Nipponbare are less affected by *S. oryzae* infestation.

Two proteins related to general metabolic processes were identified as more abundant under infested condition only in Nipponbare: CBS domain-containing protein (CBSX1) and cysteine synthase (Supplementary Table 2). In Arabidopsis, AtCBSX1 and AtCBX2 positively regulate thioredoxins, which then reduce target proteins, such as peroxiredoxin, which in turn reduce cellular H_2_O_2_ and regulate its level (Yoo et al., 2011). Recently, Kumar et al. (2018) overexpressed a rice Two Cystathionine-β-Synthase Domain-containing Protein (*OsCBSCBSPB4*) in tobacco. Transgenic seedlings exhibit delayed leaf senescence, profuse root growth, and increased biomass in contrast to the wild-type seedlings when subjected to salinity, dehydration, oxidative, and extreme temperature treatments, suggesting that OsCBSCBSPB4 is involved in abiotic stress response. As far as we know, this is the first time that a CBS protein is related to biotic stress tolerance in plants. The biosynthesis of cysteine is a limiting step in the production of glutathione, a thiol implicated in various cellular functions, including scavenging of reactive oxygen species and resistance to biotic stresses (Youssefian et al., 2001). At the same time, several studies have pointed out the function of cysteine-thiols and sulfur-based primary and secondary metabolites in basal defense responses to abiotic and biotic stresses (for a comprehensive review, see Tahir and Dijkwel, 2016). Even though herbivory resistance can employ metabolites containing cysteine such as glucosinolates (Guo et al., 2013), to the best of our knowledge, this is the first work that links the expression of cysteine synthase protein with mite tolerance.

Two proteins related to stress response were also identified as more abundant under infested condition only in Nipponbare: osmotin-like protein and ricin B-like lectin. Osmotin protein belongs to the PR-5 family of pathogenesis-related (PR) proteins, which are produced in response to various biotic and abiotic stresses. Osmotin can increase its own cytotoxic efficiency promoting changes in the cell wall structure and inhibiting defensive cell wall barriers, which in turn enables osmotin penetration into the plasma membrane (Hakim et al., 2018). Overexpression of genes encoding osmotins confers tolerance against biotic and abiotic stresses in different plant species (Barthakur et al., 2001; Xue et al., 2016; Chowdhury et al., 2017), besides stimulating the accumulation of proline, which functions as a compatible osmolyte useful in responding to abiotic stress, especially drought and salinity (Hakim et al., 2018). Proline also functions suppressing free radicals and preventing chlorophyll degradation in vegetative cells, in order to maintain the photosynthetic processes (Barthakur et al., 2001; Chowdhury et al., 2017). In the mite-tolerant Nipponbare, we detected higher levels of proline accumulation under infested condition when compared to control (Figure 9), along with maintenance of chlorophyll concentration (Figure 4) after mite infestation. This is the first time that expression of an osmotin protein is related to mite tolerance. Since the generation of transgenic plants overexpressing osmotin genes is being recommended as a biotechnological tool to generate stress tolerance, we are currently overexpressing and knocking out *OsOSM1* gene (LOC_Os12g38170) in rice plants.

Ricin B-like lectins, one of the most widespread families of carbohydrate-binding proteins (Lannoo and Van Damme, 2014), have been suggested to play a role in plant defense against pathogens (Vandenbussche et al., 2004) and insects (Wei et al., 2004; Shahidi-Noghabi et al., 2009). Since mutation of the carbohydrate-binding site can abolish or reduce the toxic effect, the entomotoxic properties of the proteins can be linked to their carbohydrate-binding activity (Shahidi-Noghabi et al., 2008). Our work is the first one that correlates a ricin B-like lectin expression with mite response/tolerance in plants. Curiously, it was shown that β-trefoil structure enables interactions between fungal ricin B-like lectins and PIs, and that these interactions modulate their biological activity (Žurga et al., 2015). It would be interesting to test whether the two PIs (putative Bowman Birk trypsin inhibitor) we found more abundant only in the mite-tolerant Nipponbare under infested condition (Supplementary Table 2) are able to interact with the ricin B-like lectins.

Among plant defenses, PIs have direct effects on herbivores, interfering with their physiology (Diaz and Santamaria, 2012; Martinez et al., 2016; Arnaiz et al., 2018). Once ingested by the arthropod, PIs inhibit proteolytic activities, preventing or hindering protein degradation (Terra and Ferreira, 2005). The first successfully overexpressed Bowman-Birk family PI gene in tobacco was the cowpea trypsin inhibitor (*CpTI*—Hilder et al., 1987). After, the *CpTI* gene was inserted into the genome of several plants, increasing the resistance to different herbivore species (Santamaria et al., 2012). Since then, different PIs have been obtained from cultivated plants such as rice, barley, soybean, sweet potato, and corn, and have been overexpressed in different plant species, conferring resistance to various pest insects (Clemente et al., 2019). Rice has seven Bowman Birk trypsin inhibitor genes, and overexpression of *RBBI2-3* in transgenic rice plants resulted in resistance to the fungal pathogen *Pyricularia oryzae*, indicating that PIs confer *in vivo* resistance against the fungal pathogen, and might play a role in the defense system of the rice plant (Qu et al., 2003). Recently, Arnaiz et al. (2018) verified that Kunitz trypsin inhibitors (KTI) participate in the plant defense responses against the spider mite *Tetranychus urticae*. Thus, our work is the first one to suggest that Bowman Birk trypsin inhibitors may be involved in the tolerance of rice plants to *S. oryzae* mite infestation.

**FIGURE 10:**
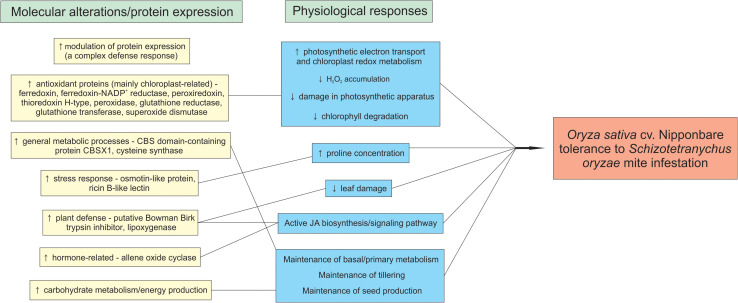
Rice mechanisms employed by *Oryza sativa* cv. Nipponbare to tolerate *Schizotetranychus oryzae* mite infestation.

The model in Figure 10 summarizes the rice tolerance mechanisms putatively employed by Nipponbare plants.

## Conclusion

Tolerance is a sustainable pest management strategy as it only involves plant response, and therefore does not cause evolution of resistance in target pest populations (Peterson et al., 2017; Sperotto et al., 2018a). According to Gerszberg and Hnatuszko-Konka (2017), elucidation of the tolerance mechanisms at the biochemical, physiological, and morphological level remains one of the greatest challenges of contemporary plant physiology. Our physiological results showed that African rice species (wild *O. barthii* and cultivated *O. glaberrima*) and weedy rice (*O. sativa* f. *spontanea*) do not present tolerance to *S. oryzae* mite infestation. Still, we characterized *O. sativa* cv. Nipponbare as tolerant to mite infestation. Proteomic data showed that *O. barthii* presents a less diverse/efficient antioxidant apparatus under infested condition, not being able to modulate proteins involved in general metabolic processes and energy production, resulting in damage to its photosynthetic apparatus and development. On the other hand, Nipponbare presents more efficient antioxidant mechanisms that maintain cellular homeostasis and its photosynthetic capacity. Tolerant plants are able to modulate proteins involved in general metabolic processes and energy production, maintaining their development. In addition, these plants can modulate the expression of defense proteins, such as osmotin, ricin B-like lectin, and PIs, which may be used in future breeding programs for increasing rice tolerance to mite infestation.

## Data Availability Statement

All proteomics data were deposited to the ProteomeXchange Consortium (Deutsch et al., 2017) via the PRIDE repository (https://www.ebi.ac.uk/pride/; Perez-Riverol et al., 2019) and can be accessed using the code PXD020940.

## Author Contributions

JS, FR, and RS conceived and designed the research. GB, ÉB, TL, JA, and AB conducted the experiments. VS and ML contributed with analytical tools. GB, ÉB, JA, AB, VS, and RS analyzed the data. GB and RS wrote the manuscript. All authors read and approved the manuscript.

## Conflict of Interest

The authors declare that the research was conducted in the absence of any commercial or financial relationships that could be construed as a potential conflict of interest.

## Footnotes

1 https://www.rgrc.dna.affrc.go.jp/

2 https://www.uniprot.org/proteomes/UP000059680

3 https://www.ebi.ac.uk/pride/

## Supplementary Material

Supplementary Table 1 Differentially abundant proteins in mite susceptible *Oryza barthii* (control × early infested condition).

Supplementary Table 2 Differentially abundant proteins in mite-tolerant *Oryza sativa* cv. Nipponbare (control × early infested condition).

## Abbreviations

DAB: diaminobenzidine
EI: early infested
GA: gibberellin
JA: jasmonic acid
LI: late infested
PI: protease inhibitor.
